# Annual Address

**Published:** 1876-05

**Authors:** W. H. Goddard

**Affiliations:** Louisville, Ky.


DENTAL REGISTER.

VOL. XXX.] MAY, 1876. [NO. 5.

### ANNUAL ADDRESS.

BY W. H. GODDARD, LOUISVILLE, KY.

*At the Commencement in the Ohio Dental College, March 2, 1876.*

GENTLEMEN, TRUSTEES, FACULTY, GRADUATES AND VISITORS:

My acceptance of the very kind and earnest invitation of the Faculty of the Ohio Dental College, to deliver the annual address on this occasion, and my presence to make an attempt to discharge the duty imposed, furnish the best evidence of my high appreciation of the honor conferred and the pleasure it will afford me.

It is doubtless expected of the speaker, on such an occasion, that he will address his remarks to those only who are about to receive the honors of the institution, and go forth from its walls to enter upon the realities, encounter the trials and enjoy the rewards of a successful professional career.

But I think it proper, while mindful of the special duty implied, to crave your indulgence if my remarks should be more general in their application.

I am well aware of the fact that I occupy the position and discharge the duty of gentlemen hitherto selected for their distinguished professional eminence, general intelligence, elegant diction, and graceful delivery, whose success in pleasing and instructing their audiences, merited the applause which they received. How marked will be the contrast between their smoothly rounded periods, sparkling with the glowing gems of thought, and the commonplace expressions of one who lays no claim to general scholarship and has no experience as an orator.

Without further apology, I shall present what I have to say to your attention, begging you to place to my credit the earnest integrity of my purpose, if I should fail to interest and instruct you.

To me, gentlemen of the graduating class, has been assigned the pleasant duty of addressing a few remarks, before you take leave of your Alma Mater, and go forth to enter upon your professional life.

It may be, notwithstanding the frequent premonitions of your teachers, that some of you, when the honors of the College shall have been awarded you, may for the time forget that your dental education is not complete.

You may possibly imagine that it will be necessary only to select a locality and enter upon the practice of professional duties. feeling assured that your diploma beautifully framed and conspicuously hung upon your office walls, will prove a passport to public favor and patronage.

My young friends, let no such glowing prospect deceive you.

Let it be granted that you have become fully acquainted with the contents of your text books, that you have thoroughly digested the lectures and can repeat the manipulations of your teachers, that you possess the advantages of cultivated scholarship and the graces of a symmetrical moral character—the last by no means the least essential—and it does not follow that your success is assured.

Men have entered every profession in life with similar qualifications possessed in a high degree, and they have ingloriously failed. Others, endowed by their Creator with no greater genius, who never studied in the dim light of the cloistered college, who never listened to learned lectures from the professor's chair, or pored over the pages of great authors in the crowded alcoves of vast libraries, persevering efforts for self-culture, have written their names indellibly upon the imperishable scroll of fame and listed them forever among the benefactors of their race.

It is vain for the young man to imagine that it is only necessary to be placed where books and apparatus abound, in order to be educated; as if knowledge could ever be obtained without labor; as if by a kind of magic, books could be read and their contents digested and remembered; as if all the colleges and universities in the land could ever be successful in developing mind without pains-taking mental labor. I hope that none of you have any such ideas. If you have let me assure you that you greatly deceive yourselves. Indulge me in a personal allusion. I have been in the practice of dentistry for the space of forty-six years. During this long period of more than an average lifetime, I have found it necessary to be continually studious. I have diligently employed the time, and used every facility in my power and have striven with the effort of a strong will, in order to make advancement in the science and art of our chosen profession; but I feel to-day that I have great progress to make before I reach the "summit of my hopes."

I had no advantages in the beginning of my professional life such as those you now possess. No dental colleges opened their doors in those days, and even preceptors could rarely be secured, for our profession was then in its infancy. You have enjoyed excellent advantages, under the most competent teachers—men eminent in their profession—and you will go forth from your Alma Mater with her endorsement—

the long sought for parchment—certifying that you have passed honorably through a collegiate course of study and practice in this institution, and that you are qualified, so far as its requirements demand, to practice dentistry.

But are you thoroughly qualified? Are you proficient? Have you nothing further to do? Fortunate indeed you are, young gentlemen, that you have enjoyed such advantages. You begin the practice of your profession greatly in advance of those who opened their offices in the days of my youth. You stand to-day upon a platform erected upon the basis of the observation, knowledge, skill and experience of a half century of untiring effort and unfaltering zeal on the part of the founders and builders of the profession. Hundreds of your predecessors, by dint of indomitable will, close investigation and persevering industry, have risen to distinction in our calling with far less encouragement and fewer opportunities in the beginning than those possessed by you; yet they pressed their way on, every thought engrossed in making some new discovery or perfecting some useful invention, by means of which some great practical help might be given to their professional brethren and some lasting benefit might be conferred upon suffering humanity.

You are about to go forth and assume your chosen position "In the world's broad field of battle, in the bivouac of life, armed Cap-a-pie" with the weapons which the pioneers in dentistry have furnished you, with all the improvements which skill and ingenuity could devise within the past fifty years.

A soldier may enter his country's service armed with sword and buckler and helmet, and a many shotted rifle, but he is not yet a warrior. He may have graduated with honor at the National Military Academy with a perfect understanding of the theory of war. He may be skilled in the manual of arms and be thoroughly acquainted with military tactics, but without a good degree of practice in the field, he is unfitted to discern and thwart the strategy of the enemy, and is not qualified to lead the rank and file to victory.

So it is with the pursuits of business and professional life.

Theoretical knowledge is of primary necessity; and yet without constant practical application and consequent material results, its possession is of comparatively small value. These results are obtained in our profession by a constant observation of what is done by others, untiring attention to business and a determination to excel in manipulative skill as well as in excellence of work.

Observe the man devoted to his vocation, whose soul is absorbed in study, investigation and experiment with a view to secure something new in theory or practice. How hopeful he is! How closely he watches, how earnestly he labors, and with what avidity he grasps every opportunity favorable for the accomplishment of his purpose.

Such is, or ought to be, the practical result of a preparatory education in our professional schools. It is the nature of genuine professionol education to accomplish this. Its end and aim ought surely to be to fully prepare the graduate to carry out an earnestly begotten desire for thorough self-culture in after life, for it must not be forgotten that the largest amount of knowledge that has been most valuable to the world, has been the result of self-reliance, self-training and general self-education. I would not be understood, certainly on such an auspicious occasion as the present, as undervaluing the College; yet it is vain for the graduate to imagine that it is only necessary to pass through a professional course of study within its walls, in order to be thoroughly possessed of a knowledge of professional duties. "There is no royal road to learning." The toiling traveler upon its rugged, ascending grade is successful only by the aid of time, energy and patience.

To such a man books are helps, but not his dependence. The vast laboratory of nature throws open her doors to him. In search of her secrets he scales her loftiest mountain summits and penetrates the depths of her darkest caves; he wanders by the banks of her murmuring streams, rippling along over the smooth stones; he rests under the shade of her grand old forest trees, and visits the quiet solitudes seldom trod by human feet. Such a student gains the most valuable information; it is that knowledge which forms the basis of the most useful books; it is the result of observation, experiment and reflection.

It is true that obstacles to progress lie in the path of such a man; but they are such only as overcome the indolent or timid mind, while before the resolute soul they vanish as they are bravely approached. Indeed when difficulties are thus bravely met, they are easily overcome. Active industry is the battery which always vanquishes them. Laziness is continually crying, "There is a lion in the way," and some one has well said that "the lion very well knows on which street indolence lives, and he is always sure to be there." What matters it, my young friends, that some of you may be of humble origin, up to this period among the children of honorable poverty? Have you thus far in life been confronted at every turn with conflicting circumstanees? Have you often become discouraged amid your painful efforts to succeed and have been tempted to turn aside into easier paths?

If you be true hearted, you need not despair. Learn to estimate your gains as far greater than your losses and your conflicts as far more valuable than your defeats are disastrous. Your very poverty is to be most highly valued as the means of teaching some of the most useful lessons of life.

You need scarcely a word of encouragement from me; yet I hail you with fraternal voice and would cheer you on in your chosen sphere. I congratulate you over difficulties already surmounted and assure you that the same unyielding fortitude will conquer thousands more. "Despise not small beginnings." Wealth, education and fame are things of growth, almost imperceptible; yet they are certain to follow if the mind bend all its energies in the work of accomplishment.

The grand old monarch of the forest that bends to the force of the hurricane and howls back the roar of the tempest, is but the development of a little seed; so the Washingtons, and Jeffersons, and Frnaklins, and Morses, and Fox, and Koecker, and Hudson, and Harris, and coming down to the present time, Arthur, Flagg, Atkinson, our own Taylor and Taft, and a host of others, who have illuminated every pathway of patriotism, statesmanship, science and dentistry, were plodding, patient men, gathering up the very elements of knowledge.

Then take you heart. By time and growth, the acorn becomes the oak.

There is ample opportunity for the exercise of genius and industry in the walks of your chosen profession.

Dentistry has by no means attained its majority. It may have passed its periods of infancy and childhood, but it is still growing and expanding in the glow of its youth; and it will continue to advance as an art and a science, until, in the vigor of maturity, its practitioners will be able to surmount every difficulty, prevent the encroachment of disease, in a great measure, if not entirely, upon the dental organs, and fully restore the imperfect to health and usefulness.

The time has already come, when the dentist must become thoroughly versed in pathology and therapeutics, for the more successful discharge of his office duties, and the period is already approaching when his scientific attainments and medical advice, in his department, will be as generally sought by intelligent parents in the rearing of their children, as those of the family physician.

Our motto, then, should be "Progress." Great responsibility rests upon us; and if you fail to enter upon the discharge of your professional duties without considering these truths, I can not predict for you success.

We have enough unworthy men in the profession, who toil only for the "almighty dollar," without regard to the honor and advancement of their calling or the comfort and health of their patients.

Our field of effort is large. The realm of knowledge is infinite in extent. Man's powers, however, are finite. As the result of his most diligent intellectual efforts, he gathers but a few pebbles upon the shore of the limitless ocean of thought.

How necessary then, that we seize every opportunity for improvement, and seek advancement by all legitimate means.

If we passively fold our arms and idly sit in our offices, with the vain idea that we have made the grand achievement, when we have secured a diploma, we greatly wrong ourselves, and encourage the studious, persistent worker, to rise above us in the professional ascent, and press onward and upward to nobler heights, while we remain amid the mist and darkness of the vale below. In short my friends, the only way to reach excellence in any calling, is continued, unremitting intellectual effort. By this I do not mean that recreation must be neglected, for it is essential to vigor of mind and health of body. Let both be properly combined, neither overtaxing the brain, nor idling precious moments in seeking pleasure for itself alone, but using both for the full development of mind and muscle.

Let a portion of time be regularly devoted to reading—history, literature and science offer abundant material for mental and moral nourishment; the writings of poets, novelists, philosophers and divines are all necessary for the development of mental activity and strength.

By no means be discouraged if every subject be not at once comprehended. Due reflection will render ideas at first occult, intelligible and instructive. There are many ways in which a mind, intent on gaining knowledge, may be improved:

1st. The habit of listening to those who have spent a long life in efforts for the advancement of sience—men who have burned the midnight lamp and spent many a sleepless hour in the solution of some intricate problem, which, though solved with great labor, is freely given to the world.

2d. Reduce to practice whatever may be learned. Expect not that every trial will be successful. Mark all failures and search for their causes, for by so doing comes frequently the best mental discipline.

Be diligent, in season and out of season, and zealously strive to excel in all pertaining to your vocation. Be not easily discouraged if success should not immediately crown your efforts.

Be patient and exercise self-control. In your practice be tender hearted and gentle in every movement. While at the same time you are thorough in every thing you do. In short, conduct yourselves toward your patient as gentlemen and act always as honest men, and ere long your name, reputation and character will be fully established and success will be your reward.

One great difficulty in our profession is that so few have been thoroughly educated. Many have never enjoyed the advantages which surround the student of the present day; they have, perhaps, failed to embrace opportunities offered them for enlarging their minds, and storing them with medical as well as dental knowledge; for in order to become a successful dentist, the student should be well up in general medical learning, and be particularly proficient in the science of anatomy.

A knowledge of all the branches of medical science makes the best foundation for the superstructure of dentistry; he who patiently lays this foundation will be the most successful practitioner, provided always, that skill in his fingers equals the activity of his brain.

A man may possess a mind stored with useful knowledge upon all subjects, but if he be comparatively destitute of manipulative skill, he can never make a thoroughly accomplished dentist.

People will soon ascertain who has delicacy of touch and performs operations with the least pain, who handles his instruments with dexterity and precision, who, apparently having eyes in his fingers, performs his work without puffing and blowing as if he were grubbing in a hazel field. I am aware that it is no easy task to stand at the chair and work over half a dozen patients during as many hours, for I have tried it often and speak from experience.

Cleanliness is an essential quality in a dental office. Avoid every thing of a disagreeable or unpleasant character.

Young gentlemen, how inestimably valuable is the present period of your lives! Now the blood of youth is bounding through its channels; now the emotions are fresh and strong; now habits are easily formed—habits which for good or ill, rivet round the whole future life—this period is now yours.

You are thus blest with the earlier energies of life—are you willing to bring them and consecrate them upon the altars of virtue, knowledge and morality? Remember how fleeting are the years of this precious period.

Change, slow, sure, though imperceptible is stealing over us all; it is stealing over you. Heed the moral lessons it silently imparts: "What thou doest, do quickly." Well hath the poet said:

"No eye perceives our growth or our decay;
To-day we look as we did yesterday.
And we shall look to-morrow as to-day,
Yet while the loveliest smiles, her locks grow gray,
And, in her glass could she but see her face.
She'll, see as soon amid another race.
How would she start!"

What a glorious destiny may await each one of you as you leave these halls, amid the opening scenes of this grand Centennial year? How mean it will be for you to live an aimless life and lie down at last in some obscure grave, to be remembered only as one who lived alone for self.

How hallowed the name and fame of him who—

"Departing, leaves behind him,
Footprints on the sand of time.
Footprints, which perhaps another,
Sailing o'er life's solemn main—
A forlorn and shipwrecked brother,
Seeing, shall take heart again."

Young friends, remember that while a soul without knowledge is dark and sad, a soul without truth and virtue is gloomier still.

Wherever the name of our immortal Clay is known and revered, his God-like utterance "I would rather be right than be President," is applauded and cherished. Let your motto be the same. Let your petition to Heaven be: "Let me be right." Let this be your perpetual idea, and may this prayer be translated into each one of your lives.

Gentlemen of the Faculty, I hope I may be pardoned, if presuming upon my age, I speak a few friendly words to you, not that I think myself your equal in the exalted relation you sustain to this institution, but because I have been for many years with many of you both in a professional and social capacity. For some time past, you have had charge of the professional education of these promising young gentlemen, who now, having passed the requisite examination, have presented themselves for your certificate of approval. In their diplomas you announce to the world that they have attained sufficient knowledge to commence the practice of their profession. They have no doubt been assiduous in their studies, prompt at the lectures, respectful to you. If such be the case, may I not ask, have you been faithful to them? Have you devoted your energies to promote their highest good, not only in imparting a knowledge of dental science, but have you set an example in your daily walk and conversation of that purity of character, that unblemished reputation, that sound morality, which should characterize true manhood?

May they safely emulate your example?

The true character of a teacher unconsciously manifests itself in his ordinary words and deeds. It is this unconscious influence that is the paramount moral force in the school, giving it its prevailing tone, and constantly moulding the characters of the students.

Above all things, therefore, it behooves the teacher to be of such a spirit that his impulses and principles shall all be pure and true. No teacher can expect his pupils to be more courteous or honorable than he is himself. He must be what he would have his students become.

Have you endeavored to excite a feeling of self-reliance and an emulative energy that shall impel to vigorous self-work? Have you labored to form the habit of keeping the observing powers in ceaseless activity, to excite, if possible, a permanent and irrepressible spirit of original investigation?

Of little comparative power and influence is the possession of superior ability, if the faculties have not been so trained as to be brought to bear on subjects of importance with promptness, vigor, clearness and logical discrimination.

From my long and intimate acquaintance with you, I believe you have performed every duty as teachers that could possibly be required, and that the young gentlemen before me, can never revert to the time spent in their collegiate course here, and truthfully assert that the Faculty of this College have been remiss in their duties. I believe that all will join with me in the just commendation, "Well done good and faithful servants." May your lives be long spared and may you reap still greater rewards for your untiring labors, in this noble institution.

And now my brethren in the profession, permit me before I close, to say a few words to you, Some of us have been scores of years devoted to this specialty. The sands of life are fast sinking with many of us. What have we accomplished in our time for the benefit of each other? What great original achievement, thought or instrumentality have we sent into the world? Much in the way of literature, development of sience, invention of appliances to aid and assist in perfecting work, relieving pain and the saving of teeth, has been accomplished. Progress has been wonderful in the past ten or twenty years. Much greater advancement is destined ere long to be made. None of us dare predict what will take place in the next fifty years.

We have just commenced the Centennial year. How few of us have thought what has been accomplished in the century whose closing has lately been celebrated. Need I speak of the American Revolution, with the birth of a new and powerful nation; the French Revolution with its influence upon all European life and political ideas; the wars of Napoleon, with their upheavals and reconstruction of nations and dynasties; the European wars of 1842; the British Emancipation in the West Indies; our late civil strife with its abolition of slavery and its establishment of our one great nationality; the French and Prussian war, with its brilliant and marvelous illustrations of the power of German military skill and discipline and many other great political and revolutionary events, which have marked the century; events with influences that have been felt throughout the world, and with consequences that will reach down to remotest ages.

Think moreover, of what science has done in the past century—chemistry, with Priestley's wonderful investigations and disclosures has become almost a new science. Electricty, as marvelous in its applications to practical ends as in its surprising unfoldings, making the lightning of Heaven the message for earth, over continents and under seas and oceans; astronomy, with telescope and spectroscope lending their keen vision and subtle analysis to its study of the mechanism of the elements of the heavenly world; geology, reading the evidences of indefinite periods of development in the rock strata of the earth—the spirit of historic research penetrating to the depths of long lost or deeply buried cities and finding the cuneiform records, and with untiring labor interpreting them and learning their stories of past monarchies and former civilization.

How transcendent have been the labors and revelations of science during this eventful century. Equally wonderful have been the activities of the spirit of inventive industry, taking nature's various powers and bidding them do in the departments of each other what the needs of an ever advancing civilization require to have done, thus adding immeasureably to the energies and results of intelligent labor.

I have referred thus briefly to some of the results which have transpired during the past century, many more could be named did time permit.

Enough have been recited to stimulate every one to greater exertion and more constant endeavor to surmount every obstacle and press on to still greater achievement.

In conclusion permit me to say, young gentlemen, that it will take time for you to secure a lucrative practice. Communities are very slow in bestowing their confidence and patronage upon young physicians and dentists. But keep up a good heart. Be dilligent. Be always at your post. Never absent yourself because you have no engagement, but utilize the time by reading or study, and ere long your reward will be abundant.

I should prove faithless to the choicest inspiration of my heart, did I fail to recognise in fitting terms the presence of those upon this occasion, who are dearer to us all, than the life blood which stirs our pulses. And in saying this I am guilty of no outburst of unmeaning compliment. The days when the utterance of extravagant sentimentalism, about the lovliness of women, was a frequent feature of public addresses before mixed audiences, and the chivalry of flowery comment on the "gleaming hair," the "piercing eye," the "ruby lips," and the "bewitching smiles," of the fair, was thought to more than redeem its silliness, have passed away. Public opinion is demanding in behalf of women, that those powers of her mind, and those refining graces of her character, which are vital forces in human society, shall be so fully and considerately recognised, as to make such shallow prettinesses of address to her beauty, as once were current, as unbecoming for man to utter as they are distasteful to women to hear. A nobler range of encomium is deserved and is demanded, And as I say this I am stirred by knowing a majority of those, with whom the dentists are daily associated professionally, are mothers, wives and daughters, who manifest much greater strength of mind, purpose and endurance, than the so called sterner sex.

What would we be, were it not for the attraction and persuasive qualities of women, the light and life of our hearts and homes?

Therefore, I bid hearty welcome to the fair who have honored us by their presence, not as mere spectators, who are the mere recipients of one gratuitous courtesy, but as those from whose lives and characters we derive, in part, the inspirations of duty and power to our progressive endeavors.

We tender them more, far more than chivalrous courtesy. They have our acknowledged deference—our profound respect, aye, our sincere love, and speaking in such a spirit, welcome, thrice welcome, to them all.

